# Collective behavior of soft self-propelled disks with rotational inertia

**DOI:** 10.1038/s41598-022-26994-2

**Published:** 2022-12-29

**Authors:** Soumen De Karmakar, Anshika Chugh, Rajaraman Ganesh

**Affiliations:** 1 Institute for Plasma Research, Bhat, Gandhinagar, 382428 India; 2 Homi Bhabha National Institute, Training School Complex, Anushaktinagar, Mumbai, 400094 India

**Keywords:** Condensed-matter physics, Statistical physics, thermodynamics and nonlinear dynamics, Biological physics, Soft materials

## Abstract

We investigate collective properties of a large system of soft self-propelled inertial disks with active Langevin dynamics simulation in two dimensions. Rotational inertia of the disks is found to favor motility induced phase separation (MIPS), due to increased effective persistence of the disks. The MIPS phase diagram in the parameter space of rotational inertia and disk softness is reported over a range of values of translation inertia and self-propulsion strength of the disks. Our analytical prediction of the phase boundary between the homogeneous (no-MIPS) and MIPS state in the limit of small and large rotational inertia is found to agree with the numerical data over a large range of translational inertia. Shape of the high density MIPS phase is found to change from circular to rectangular one as the system moves away from the phase boundary. Structural and dynamical properties of the system, measured by several physical quantities, are found to be invariant in the central region of the high density MIPS phase, whereas they are found to vary gradually near the peripheral region of the high density phase. Importantly, the width of the peripheral region near the phase boundary is much larger compared to the narrow peripheral region far away from the phase boundary. Rich dynamics of the disks inside the high density MIPS phase is addressed. Spatial correlation of velocity of the disks is found to increase with rotational inertia and disk hardness. However, temporal correlation of the disks’ velocity is found to be a function of rotational inertia, while it is independent of disk softness.

## Introduction

Interesting collective behavior^1–15^ is observed over every scale of active matter, owing to their persistent self-propulsion. One such self-organizing property is the formation of clusters of high and low density phase in a homogeneous system of uncoordinated self-propelled particles. Phase coexistence of high and low density phase in active or self-propelled system is known as motility induced phase separation (MIPS)^16^, as MIPS is only observed in motile or active system. MIPS has been investigated both in overdamped^17–19^ and underdamped^20,21^ limits. However, in standard numerical models, sufficiently hard particles^17–20,22,23^ are frequently explored. Solid-like high density MIPS clusters are observed in the hard particle limit^17,21,24,25^, as particles are less deformed. Recently, liquid-like high density MIPS state has been found in a collection of low density self-propelled particles that align their self-propulsion direction along the inter-particle separation and towards the nearest neighbors^26–28^. Although, a system of soft active particles, as in most biological active matter, is addressed in some of the recent studies^21,29^, a detailed investigation of the collective structural and dynamical properties in the high and low density MIPS state for soft self-propelled particles is yet to be addressed.

Rotational inertia is an important aspect in inertial active systems^21,30–35^. For instance, it has been found to introduce delay between the velocity and the self-propulsion direction of the self-propelled particles and influence their long time dynamics^36^. Role of rotational inertia on MIPS of hard self-propelled disks has been recently addressed^37^. Rotational inertia can be controlled by movement of specific limbs in biological active matter. Animals, such as Cheeta, control their stability during fast runs by moving their tail^36^. The general strategy of variation in dynamic mass distribution^21,38^ is used in practice to control rotational inertia in artificial active matter. In spite of its crucial role in controlling the self-propulsion of active particles, a detailed investigation of rotational inertia on the structure and dynamics of MIPS states is an open problem.

We explore the combined effect of particle softness and rotational inertia on the self-organized collective properties of self-propelled particles employing Langevin dynamics simulation in two dimensions. Due to the two dimensional nature of the problem, we call the self-propelled particles as self-propelled disks. We show the MIPS phase diagram in the space of rotational inertia and disk softness. Our analytical predictions of the phase boundary match the numerical data remarkably well. Structural and dynamical properties of the various regions of the phase space is investigated. For example, we show that the structural and the dynamical properties are constant in the central region of the high density MIPS state, whereas it varies gradually in the peripheral region near the phase boundary. Narrow peripheral region is found to exist in the system that is away from the phase boundary, as in overdamped MIPS clusters^5,17^. Most importantly, a thick peripheral region is found for the system near the phase boundary. Distinctive features in time evolution of the system in various regions of our phase space is addressed. Furthermore, spatio-temporal behavior of the velocity and the self-propulsion direction of the disks are investigated. At this point, we emphasize that our results are novel, and the previous studies on soft disks and rotational inertia, individually, can not be combined together to obtain the results demonstrated in this work. In “Model and Methods” Section, we introduce the model. Results are shown and discussed in “Results and discussion” Section. Finally, the conclusions are drawn in “Conclusion” Section.

## Model and methods

We perform Langevin dynamics^30^ simulation on *N* = 48,400 self-propelled disks in a commensurate rectangular box of dimensional ratio $L_x / L_y = 2 / \sqrt{3}$ with periodic boundary conditions using GPU and CPU versions of the Molecular Dynamics solver MPMD^21,27,39,40^. Each disk with diameter $\sigma$, mass *m*, and moment of inertia *I* interacts with other disks in the system through a modified Yukawa potential^21,27^, such that the total conservative potential is $U = U_0 \displaystyle {\sum _{i<j} e^{-(r_{ij}-\sigma )/ \lambda }/r_{ij}}$. $U_0$ is the interaction strength and $\lambda$ controls the softness of the disks. Dynamics of the center of mass ${\textbf {r}}_i$ and the self-propulsion direction $\theta _i$ of the disks are governed by the set of *N* active Langevin equations:

1 $$\begin{aligned} m \dot{{\textbf {v}}_i}&= -\gamma {\textbf {v}}_i + {\textbf {F}}^{I}_{i} + {\textbf {F}}^{a}_{i}, \end{aligned}$$

2 $$\begin{aligned} I \ddot{\theta }_i&= -\gamma _r {\dot{\theta }}_i + \sqrt{2 \gamma _r^2 D_r} \xi _i(t). \end{aligned}$$

We have neglected thermal noise of the translational motion in this study, as the thermal noise is orders of magnitude smaller^22,34,41^ compared to the other forces in Eq. (1). The suffix *r* denotes rotational parameter. $\gamma , \gamma _r$ are friction coefficients. $D_r$ is the angular diffusion coefficient of the direction of self-propulsion ${\textbf {n}}_i = (\cos \theta _i , \sin \theta _i)$. $\xi$ is the Gaussian white noise with zero mean and unity variance. ${\textbf {F}}_i^I$ is total conservative force on disk *i*, such that ${\textbf {F}}_i^I = -\varvec{\nabla }_i U$. The self-propulsion force ${\textbf {F}}_i^a$ is responsible for the motility of the disks. The disks move persistently with constant speed $\text {v}_0$ along the self-propulsion axis ${\textbf {n}}_i$ for an average time $\tau _p = 1/D_r$. The average time $\tau _p$ is the persistence time of the disks. We consider $\sigma$ and $1/D_r$ as the normalized unit for length and time, respectively. Energy is measured in the unit of $m \sigma ^2 D_r^2$. The normalized dynamical equations are:

3 $$\begin{aligned} M \dot{{\textbf {v}}_i}&= -{\textbf {v}}_i - \varvec{\nabla }_i \Gamma \sum _{i < j} \frac{e^{-\kappa (r_{ij}-1)}}{r_{ij}} + \text {P}_e {\textbf {n}}_i, \end{aligned}$$

4 $$\begin{aligned} J \ddot{\theta }_i&= -{\dot{\theta }}_i + \sqrt{2} \xi _i(t). \end{aligned}$$

$M = \frac{m/ \gamma }{1/D_r}$ and $J = \frac{I/ \gamma _r}{1/D_r}$ are the reduced translational and rotational inertia, respectively. Strength of activity of the disks is controlled by the Peclet number $\text {P}_e = \frac{\text {v}_0 / D_r}{\sigma }$. In normalized units, Peclet number is the ratio of persistence length $\text {v}_0 / D_r$ to the diameter $\sigma$ of the self-propelled disks. Hence, the Peclet number scales with the activity of the disks. $\Gamma = \frac{U_0}{\gamma \sigma ^3 D_r}$ and $\kappa = \frac{\sigma }{\lambda }$ are the reduced interaction strength and softness parameter of the disks, respectively. With decrease in $\kappa$, softness of the disks increases^21^, whereas hardness increases with increase in $\kappa$.

Area fraction $\phi = N \pi / 4 L_x L_y$ is fixed at 0.65, as MIPS is observed without rotational inertia for the considered value of $\phi$. We consider constant interaction strength $\Gamma = 25$, which is found to be an optimum value for the complete range of softness parameter $\kappa$ in this work, as the value above and below $\Gamma = 25$ shifts the MIPS phase boundary towards higher $\kappa$, similar to that in Ref. 21^21^. Interaction cut-off is set to $r_c = 8.0$, which is significantly large compared to the range of inter-particle interaction of the particles. Integration time step $\delta t$ is set to $10^{-4}$.

## Results and discussion

Several interesting collective properties of the self-propelled inertial disks are provided and discussed below.

### MIPS phase diagram

**Figure 1:**
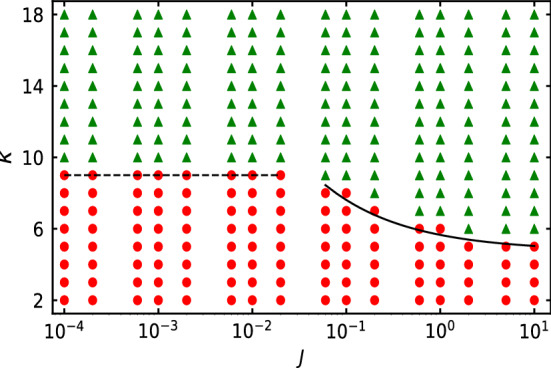
Phase diagram in $J-\kappa$ space for $M = 0.05$ and $\text {P}_e = 125$. Green (triangles) and red (circles) markers denote MIPS and homogeneous phase respectively. Dashed and solid lines are the analytic expressions for the phase boundary, provided in Eq. (6) in the limit of small and large *J*, respectively.

The disks are distributed almost homogeneously in the limit of large softness ($\kappa \rightarrow 2$). Nearly homogeneous distribution of the disks is called as homogeneous phase. At sufficiently large value of softness parameter $\kappa$ and rotational inertia *J*, the self-propelled system is found to phase separate into a high density and a low density region, that coexist. The coexistence of high and low density region is known as MIPS. In Fig. 1, the phase diagram of our system of soft self-propelled disks in the space of rotational inertia *J* and softness parameter $\kappa$ in semi-log scale for fixed translational inertia $M = 0.05$ and Peclet number $\text {P}_e = 125$ is shown.

Green (triangles) and red (circles) markers denote, respectively, the MIPS and the homogeneous phase. MIPS state in the phase diagram is obtained from two distinct peaks in the distribution of local area fraction. In the limit of small values of *J* ($J < 0.06$), the homogeneous system of soft disks is found to exhibit MIPS at sufficiently large $\kappa$ ($\kappa > 9$). In the small *J* limit, the phase boundary between the homogeneous and the MIPS state is independent of *J*. In the limit of large values of *J* ($J \ge 0.06$), the collection of self-propelled disks are found to exhibit MIPS at significantly small values of $\kappa$ or large softness. In the limit of large *J*, phase boundary monotonically shifts towards small $\kappa$ with increase in *J*. Unlike softness and translational inertia, which in general oppose MIPS, rotational inertia is found to favor MIPS such that MIPS is observed for extremely soft disks with increase in rotational inertia.

To understand the phase diagram, more specifically the shape of the phase boundary, a time scale $\tau _{\kappa }$ is associated to the deformation of the soft disks. The softness timescale $\tau _{\kappa }$ is defined as $\tau _{\kappa } = \tau _{P} / \kappa$. The hard disks at large $\kappa$ are less deformed despite persistent collision with the other disks. Consequently, $\tau _{\kappa }$ is small for hard disks, whereas the soft disks at small $\kappa$ deform significantly, that in turn increases $\tau _{\kappa }$. Moreover, $\tau _{\kappa }$ increases with Peclet number $\text {P}_e$, as the soft disks are deformed more with increase in $\text {P}_e$. As the deformation of the disks increases with increase in $\tau _{\kappa }$, the effective size of the two-particle-cluster becomes smaller. Interestingly, with increase in *J*, temporal correlation of the self-propulsion direction ${\textbf {n}}_i$ increases, as shown in the inset of Fig. 7a. Hence, the effective persistence time $\tau _{P}^e$ of the self-propelled disks increases with increase in rotational inertia *J*. In the limit of small and large *J* , that is in the limit of small rotational inertial time scale $\tau _I = I / \gamma _r$ with respect to persistence time scale $\tau _P$ and vice-versa,, the analytical expression of $\tau _{P}^e$ as obtained by Caprini et al.^37^ is

5 $$\begin{aligned} \tau _P^e \sim {\left\{ \begin{array}{ll} &{} (1 + J) / D_r, \quad\text {small}~ J,\\ &{} J^{1/2} / D_r^{3/2}, \quad\text {large}~ J. \end{array}\right. } \end{aligned}$$

The system of soft self-propelled disks phase separate when the effective persistence time becomes sufficiently large such that the small two-particle-cluster remain together for sufficiently large time for the other disks to collide with the nucleation site of this two-particle-cluster. Hence, the necessary criteria to exhibit MIPS is that the softness time $\tau _{\kappa }$ of soft self-propelled disks should scale with the effective persistence time $\tau _P^e$, that is $\tau _P^e \sim \tau _{\kappa }$. Hence, the scaling of the phase boundary in the appropriate limit of *J* is obtained as

6 $$\begin{aligned} \kappa \sim {\left\{ \begin{array}{ll} &{} 1 - J, \quad\text {small}~ J,\\ &{} J^{-1/2}, \quad\text {large}~ J. \end{array}\right. } \end{aligned}$$

In Fig. 1, the analytical expression for the phase boundary in the limit of small and large *J*, respectively, are shown by the dashed and the solid line. Our analytic prediction Eq. (6) fits well with the simulation data.

As translational inertia *M* suppresses and Peclet number $\text {P}_e$ favors MIPS, the phase boundary between the homogeneous and MIPS states in the $J-\kappa$ space is found to shift towards larger $\kappa$ with increase in *M* and decrease in $\text {P}_e$. In Supplementary Fig. S1, the phase diagram in the $J-\kappa$ space for a smaller value of Peclet number $\text {P}_e = 75$ and for three different values of *M*, spanning over two orders of magnitude, namely $M = 0.005$, $M = 0.05$, and $M = 0.5$ is shown. In the small *J* limit, the horizontal phase boundary shifts towards large $\kappa$ , that is towards substantially hard disks, with increase in *M* and decrease in $\text {P}_e$. With increase in $\kappa$, bounce back effect^20^ on collision of two inertial self-propelled disks becomes important. Interestingly, our analytic prediction for the phase boundary matches well with the numerical data, even with variation in *M* and $\text {P}_e$. Due to shift in the phase boundary towards large $\kappa$ with increase in *M* and decrease in $\text {P}_e$, the proportional constant factor in the analytic expression for the phase boundary should be a function of $M / \text {P}_e$. Due to similar variation of the phase boundary in the limit of large *J*, the proportionality constant should be a function of $M / \text {P}_e$. However, the exact dependency of phase boundary on the $M / \text {P}_e$ is out of the scope of this study.

### Shape of the high density phase

**Figure 2:**
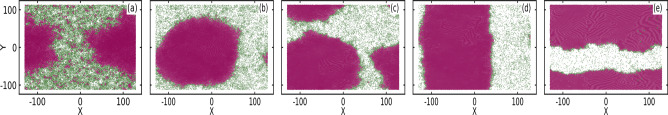
Configuration of the system for (**a**) $\kappa = 6$, (**b**) $\kappa = 8$, (**c**) $\kappa = 10$, (**d**) $\kappa = 11$, and (**e**) $\kappa = 18$. Disks in the low and high density phase are colored green and magenta, respectively. Inertial parameters are fixed at $M = 0.05$ and $J = 2.0$, and the Peclet number is fixed at $\text {P}_e = 125$. Circular clusters near the phase boundary are stretched along one of the dimensions and become rectangular with increase in disk hardness.

In Fig. 2, we have shown the configuration of the system for $\kappa = 6, 8, 10, 11, 18$ from left to right for fixed $J = 2$, $M = 0.05$, and $\text {P}_e = 125$, corresponding to the vertical column at $J = 2$ in MIPS region of Fig. 1.

If the local area fraction of the particles is greater than the considered global area fraction $\phi = 0.65$, then those particles are considered to constitute the dense cluster phase, else they are considered to constitute dilute or low density phase. Particles in the high and the low density region is colored magenta and green, respectively. The configurations are obtained at $t = 100$. Close to the phase boundary, the shape of the high density phase is found to be nearly circular, while the shape is near rectangular as the system is much away from the phase boundary. Thus, we observe the shape change from circular to a rectangular one as we move away from the phase boundary with increase in $\kappa$. It is interesting to note that the shapes of the clusters may vary^13^ with variation in the geometry of the simulation box. For simplicity and without loss of generality, in this work, we consider a rectangular simulation box of fixed dimensions. Near the phase boundary, it has been previously^21^ observed that the phase separation time increases. To justify the fact, that at large time the shape remains circular near the phase boundary, we show the configuration for $J = 2$, $\kappa = 7$, $M = 0.05$, and $\text {P}_e = 125$ at $t = 50$ (short), $t = 100$ (intermediate), and $t = 500$ (large) in Supplemental Fig. S2. At all three times the high density region is found to be near circular. The circular shape near the phase boundary is found at small as well as large inertia. We show the configuration for the parameters $M = 0.5$ (large inertia), $J = 2$, $\kappa = 18$, and $\text {P}_e = 125$, for which the system is near the phase boundary and in the MIPS state, in supplemental Fig. S4b. The high density phase is near circular despite the hard particle limit at $\kappa = 18$. The detailed analysis of the observed clusters is performed in the following Sections.

### Structural properties

**Figure 3:**
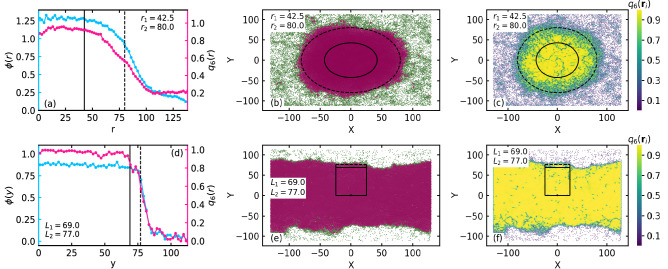
Local structural properties for $\kappa = 7$ (top row) and $\kappa = 17$ (bottom row) for fixed $M = 0.05$, $J = 2$, and $\text {P}_e = 125$. (**a**,**d**) Local area fraction $\phi$ (left vertical axis) and the magnitude of local orientational order $q_6$ (right vertical axis) from the center of the high density phase. For calculational ease, the high density phase is shifted to the center of the simulation box. The boundary of the central and the peripheral region are shown by the solid and the dashed line in (**a**,**d**) (see the main text for definition of central and peripheral region). (**b**,**e**) Configuration of the system. The disks in the high and the low density phase are colored magenta and green, respectively. Boundary of the central and peripheral region for the circular high density phase is denoted by $r_1 = 42.5$ and $r_2 = 80$, respectively, and that for the rectangular high density region is denoted by $L_1 = 69$ and $L_2 = 77$, respectively (see main text for definition of central and peripheral region). (**c**,**f**) Configuration of the system, colored according to the magnitude of orientational order $q_6({\textbf {r}}_i)$ of the disks.

Near and away from the phase boundary the local structural properties of the system exhibit very distinctive features. In Fig. 3, we have shown the local structural properties for $\kappa = 7$ (top row) and $\kappa = 17$ (bottom row) for fixed $M = 0.05$, $J = 2$, and $\text {P}_e = 125$.

In Fig. 3a,d, local area fraction^26^ $\phi$ (left vertical axis) and the magnitude of the local orientational order^21,25^ $q_6 = < |\frac{1}{6} \sum _{k=1}^{6} e^{j6\theta ({\varvec{r}_{ik}})} |>$ (right vertical axis) from the center of the high density phase towards the periphery is shown. $\theta (\varvec{r}_{ik})$ is the angle between the two disks at ${\textbf {r}}_i$ and ${\textbf {r}}_k$ with respect to the x-axis. *j* is the imaginary number, and $<...>$ denotes the local average. For $\kappa = 7$, which is near the phase boundary, and for $\kappa = 17$, which is away from the phase boundary, the high density phase is circular and rectangular, respectively. Moreover, for calculational ease the center of the high density phase is shifted to the center of the simulation box. The configuration of the system is shown in Fig. 3b,e for $\kappa = 7$ and $\kappa = 17$. The particles in the high and the low density phase are colored magenta and green, respectively. The central and the peripheral region is denoted by the area inside the solid circle (denoted by $r_1$) and the annular area between the solid and the dashed circles (denoted by $r_2$) in Fig. 3b, respectively. For the rectangular cluster, we consider the central and the peripheral region respectively by an inner and an outer rectangular box of width 50 units. Outer boundary of the central (denoted by $L_1$) and the peripheral (denoted by $L_2$) rectangular box is shown by the solid and dashed lines, respectively. Hence, for both the circular and the rectangular clusters, solid and dashed line denote the boundary of the central and the peripheral region, respectively, as in Fig. 3a,d. The boundary of the central and the peripheral region is $r_1 \approx 42.5$, $r_2 \approx 80$ for $\kappa = 7$ and $L_1 \approx 69$, $L_2 \approx 77$ for $\kappa = 17$. In Fig. 3c,f, the magnitude of the orientational order $q_6({\textbf {r}}_i)$ of the disks for $\kappa = 7$ and $\kappa = 17$ are shown. Local structural properties remain nearly constant in the central region, while they vary gradually in the peripheral region. The width of peripheral region $r_p = r_2 - r_1 = 37.5$ for $\kappa = 7$ is much larger compared to that ($r_p = 8$) for $\kappa = 17$. The magnitude of the local area fraction in the central region is sufficiently smaller for the hard disks ($\kappa = 17$) compared to that for the soft disks ($\kappa = 7$). This is due to the fact that the soft disks deform substantially, whereas the hard disks practically do not deform upon collision with the other disks. Consequently, the central region is jammed for the hard disks, while the soft disks have the liberty to infiltrate towards the center of the central region due to their large softness. Inside the central region, the magnitude of local orientational order is $q_6 \approx 1$ for the hard disks, while it is slightly smaller than 1 for the soft disks because of their softness.

**Figure 4:**
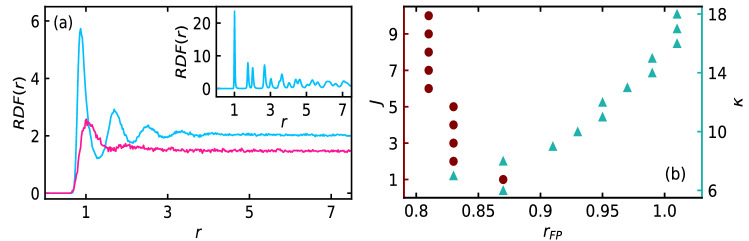
(**a**) Radial distribution function RDF(*r*) in high (blue) and low (red) density phase for the soft disks at $\kappa = 6$ for $M = 0.05$, $J = 2$, and $\text {P}_e = 125$. Inset shows the RDF for hard disks at $\kappa = 18$. (**b**) Radial location of the first RDF peak $r_{FP}$ with variation in *J* (left vertical axis) (circles) for fixed softness $\kappa = 7$ and with variation in $\kappa$ (right vertical axis) (triangles) for fixed $J = 2$, at constant $M = 0.05$ and $\text {P}_e = 125$.

Angular average of pair correlation function or the radial distribution function RDF(*r*)^21,27^ in the high and the low density phase measures the translational order of the phases. In Fig. 4a, we show the RDF(*r*) in the high (blue) and the low (red) density phases for the parameters $M = 0.05$, $J = 2$, and $\text {P}_e = 125$ for the soft disks at $\kappa = 6$.

The low density phase is clearly fluid-like as there is only a single peak in the RDF(*r*). There are few peaks in the high density phase for the soft disks at $\kappa = 6$. In the inset of Fig. 4a, we show the RDF(*r*) for the hard disks at $\kappa = 18$ in the high density phase. Not only several RDF(*r*) peaks are present for $\kappa = 18$, the first few peaks are distinctly divided into two sub-peaks, which is a characteristic of hexagonal crystal^42^ in passive two dimensional system. Moreover, the magnitude of the local orientational order of the system is nearly 1 in the central region for both the soft and the hard disks (see Fig. 3a,d). Hence, the system of soft disks resembles a two dimensional hexatic phase and the hard disks resembles a two dimensional crystalline phase^19,27,43^. The radial location $r_{FP}$ of the first peak of RDF(*r*) in the high density phase is a measure of inter-particle separation^21^ of the high density disks. In Fig. 4b, we show the variation of $r_{FP}$ with *J* (left vertical axis) (circles) for fixed $\kappa = 7$ and with $\kappa$ (right vertical axis) (triangles) for fixed $J = 2$. Inertial parameter *M* and Peclet number $\text {P}_e$ are fixed at 0.05 and 125, respectively. With increase in disk hardness (increase in $\kappa$), the inter-particle separation increases, which is consistent with the fact that deformation decreases with increase in disk hardness. Importantly, with increase in *J*, the inter-particle separation decreases by a small amount.

At small inertia $M = 0.005$, similar structural properties with wide peripheral region near the phase boundary at small $\kappa$ and a narrow peripheral region away from the phase boundary in the limit of large $\kappa$, analogous to that for $M = 0.05$, is observed. At $M = 0.005$, the structural properties and the corresponding configuration for $\kappa = 6$ (near the phase phase boundary) and $\kappa = 18$ (away from the phase boundary) are shown in Supplementary Fig. S3a,b,d,e, respectively, for fixed $J = 1$, $\text {P}_e = 125$. However, the width of the peripheral region near the phase boundary at large inertia $M = 0.5$ is found to be significantly small compared to that at small inertia ($M \le 0.05$). Importantly, with increase in *J*, the system moves away from the phase boundary, analogous to that with increase in $\kappa$. In Supplementary Fig. S4a,b,d,e, the structural properties and the corresponding configurations with increase in *J*, namely $J = 2$ (near the phase boundary) and $J = 10$ (away from the phase boundary), for fixed *M* at large value $M = 0.5$ and $\kappa = 18$ are shown. The width of the peripheral region near the phase boundary for $M = 0.5$ is $r_p \approx 20$ compared to wider $r_p \approx 38.5$ for $M = 0.05$. The disks become harder with increase in $\kappa$. Consequently, the deformation of the disks are less at large inertia *M*, compared to large deformation at small inertia *M*, due to the presence of phase boundary at relatively smaller values of $\kappa$. Less deformation is reflected in the small magnitude of local area fraction $\phi \approx 0.87$ (Fig. S4a) in the central region for $M = 0.5$, compared to $\phi \approx 1.25$ for $M = 0.05$ (Fig. 3a) in the central region. Hence, the less wide peripheral region at large inertia is due to the fact that the phase boundary, in fact, is observed at large $\kappa$.

### Dynamical properties

**Figure 5:**
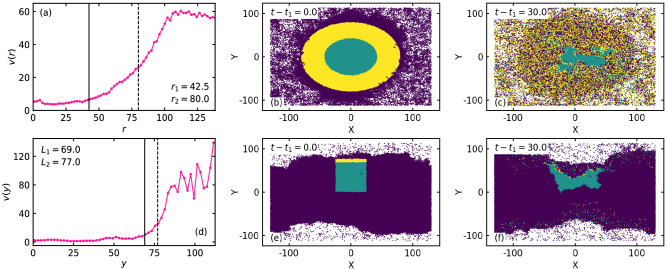
Local speed $\text {v} = \langle ({\textbf {v}}_i \cdot {\textbf {v}}_i)^{1/2} \rangle$ as a function of distance from the center of the high density phase for (**a**) $\kappa = 7$ and (**d**) $\kappa = 17$, corresponding to the configuration in Fig. 3b,e, respectively. The configuration of the system at an arbitrary initial time $t = t_1$ for (**b**) $\kappa = 7$ and (**e**) $\kappa = 17$. At $t = t_1$, the disks in the central region are colored green and that in the peripheral region are colored yellow. The other disks are colored purple. (**c**,**f**) Configurations at a later time $t = t_1 + 30$.

We show the local speed^26^ $\text {v} = \langle ({\textbf {v}}_i \cdot {\textbf {v}}_i)^{1/2} \rangle$ as a function of distance from the center of the high density phase for $\kappa = 7$ in Fig. 5a and $\kappa = 17$ in Fig. 5d.

Identical values for the other parameters are considered as that in Fig. 3. The boundaries of the central and the peripheral regions are same as shown in Fig. 3. Local speed remains nearly constant in the central region and it increases in the peripheral region. The magnitude of the local speed in the central region is more for the soft disks ($\kappa = 7$) than for the hard disks ($\kappa = 17$). At an arbitrary initial time $t = t_1$, the configuration for $\kappa = 7$ and $\kappa = 17$ are shown in Fig. 5b,e, respectively. At $t = t_1$, the disks are colored green in the central region and yellow in the peripheral region, and the other disks are colored purple. Configuration at a later time $t = t_1 + 30$ is shown in Fig. 5c,f. From the Fig. 5b,e, it is clear that the dilute phase for $\kappa = 7$ is more crowded compared to the dilute phase for $\kappa = 17$. Consequently, the values of the local area fraction is large in the dilute phase for $\kappa = 7$ (Fig.  3a) as that for $\kappa = 17$ (Fig.  3d). As a result of comparatively large area fraction or more crowding, collisions between the disks are more in the dilute phase for $\kappa = 7$ than that for $\kappa = 17$. Hence, the average magnitude of local speed for the disks in the low density phase is less for $\kappa = 7$ due to more collisions as that for $\kappa = 17$.

**Figure 6:**
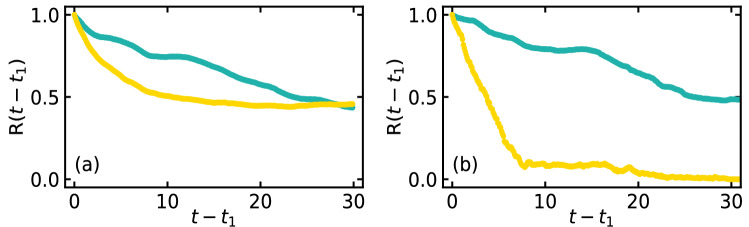
Retention fraction $\text {R}(t - t_1) = N_0(t - t_1) / N_0(t = t_1)$ for $t > t_1$ in the central (green) and the peripheral (yellow) region of the high density phase for (**a**) $\kappa = 7$ and (**b**) $\kappa = 17$. The other parameter values are same as in Fig. 5.

Number of disks in a specific region of the high density phase at $t = t_1$ is denoted by $N(t = t_1)$. Due to the dynamical movement of the disks, let $N(t - t_1)$ be the number of disks retained in that particular region, out of the initial $N(t = t_1)$ disks, at $t - t_1$. To quantify the disk movement in the high density phase, we define the retention fraction^26,27^ $\text {R} (t - t_1) = N(t - t_1) / N(t = t_1)$ for $t > t_1$, the number of disks retained at $t - t_1$ with respect to the number of disks at $t = t_1$. In Fig. 6, we show the retention fraction $\text {R}(t - t_1)$ for $\kappa = 7$ (Fig. 6a) and $\kappa = 17$ (Fig. 6b).

Green and yellow curves correspond to the particles in the central and the peripheral region, respectively, as shown in Fig. 5b,e. In the central region, the local speed becomes small for the soft disks, as the local area fraction becomes large. As the local speed for the soft disks at $\kappa = 7$ in the peripheral region is sufficiently large, the particles in the peripheral region mix throughout the system. Hence, the retention fraction of the soft disks in the peripheral region (yellow curve in Fig. 6a) saturate quickly. However, due to their softness, the peripheral disks (yellow disks) and the disks in the low density phase (purple disks) at $t = t_1$ steadily move towards the center of the high density region. Consequently, the soft disks of the central region at $t = t_1$ (green disks) steadily shuttle out of it, to maintain the constant area fraction in the central region. Hence, a finite slope is found for the green curve of $R(t - t_1)$ at $t = t_1 + 30$ for the soft disks ($\kappa = 7$). At much larger time $t - t_1 > 30$, it saturates. Time evolution for the soft disks ($\kappa = 7$) is shown in Supplementary Movie 1. On the other hand, due to extremely small deformation and negligible speed of the hard disks ($\kappa = 17$), it is practically impossible for the peripheral disks (yellow) and the disks in the low density phase to penetrate deep inside the central region. Small number of disks in the narrow peripheral region shuttle out of the high density phase and facilitate dynamic variation of the boundary of the high density phase. Hence, $R(t - t_1)$ corresponding to the peripheral disks at $\kappa = 17$ saturates quickly at small value ($R(t - t_1) \rightarrow 0$). Peripheral disks (yellow colored disks) rejoin the high density phase through the other side, due to periodic boundaries. A finite fraction of disks still penetrate inside the central region only due to dynamic variation of the boundary or the overall dynamical variation in shape of the high density region. Hence, $R(t - t_1)$ for the hard disks in the central region nearly saturates at $t - t_1 \approx 27$. Dynamical variation of the system of hard disks $\kappa = 17$ is shown in Supplementary Movie 2. In the phase separated state at small ($M = 0.005$) and large ($M = 0.5$) inertia similar dynamical properties, as for $M = 0.05$, is observed (see Supplementary Figs. S3c,f and S4c,f.

With increase in rotational inertia *J*, the local speed of the soft disks in the central regions increases, due to increase in effective persistence time $\tau _P^e$ of the disks. In spite of the increase in local packing fraction of the soft disks with increase in *J*, the peripheral disks and the disks in the low density phase at an arbitrary time $t = t_1$ move to the center of the high density phase more quickly, due to increase in local speed in the central region with *J*. In Supplementary Fig. S5, we compare the local speed (left vertical axis) and the local area fraction (right vertical axis) in the MIPS state for two different values of *J*, namely $J = 2$ and $J = 10$, for fixed $M = 0.05$, $\kappa = 7$, and $\text {P}_e = 125$. Local speed in the central region is much larger for $J = 10$ compared to that for $J = 2$. In Supplementary Movie 3 we show the dynamic evolution of the system at large rotational inertia ($J = 10$) and for fixed parameters $M = 0.05$, $\kappa = 7$, and $\text {P}_e = 125$.

### Temporal and spatial correlations

**Figure 7:**
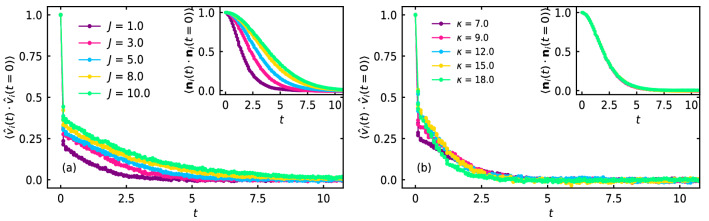
(**a**) Velocity auto-correlation $\langle \hat{\text {v}}_i (t) \cdot \hat{\text {v}}_i (t = 0) \rangle$ for various values of *J*, shown in the legend, for fixed $\kappa = 7$, $M = 0.05$, and $\text {P}_e = 125$. Inset shows the auto-correlation of propulsion direction $\langle {\textbf {n}}_i (t) \cdot {\textbf {n}}_i (t = 0) \rangle$ for the same set of *J* and the other parameters considered in (**a**). (**b**) $\langle \hat{\text {v}}_i (t) \cdot \hat{\text {v}}_i (t = 0) \rangle$ for several values of the softness parameter $\kappa$, shown in the legend, for fixed $J = 2$. Inset shows $\langle {\textbf {n}}_i (t) \cdot {\textbf {n}}_i (t = 0) \rangle$ for the same value of $\kappa$ in (b). Other parameters are same as in (**a**).

In the low density phase, the velocity of the self-propelled disks ${\textbf {v}}_i$ align with the self-propulsion direction ${\textbf {n}}_i$ of the disks, due to infrequent interaction with the other disks. However, in the high density phase, due to frequent collisions with the neighboring disks, alignment between ${\textbf {v}}_i$ and ${\textbf {n}}_i$ is found to vanish, in general. To understand the importance of rotational inertia and softness of the disks on the spatio-temporal properties, in Fig. 7a, we show the velocity auto-correlation^42^ $\langle \hat{\text {v}}_i(t) \cdot \hat{\text {v}}_i(t = 0) \rangle$ of the disks for several values of rotational inertia *J*, shown in the legend, for fixed $\kappa = 7$, $M = 0.05$, and $\text {P}_e = 125$.

$\hat{\text {v}}_i$ implies ${\textbf {v}}_i / |{\textbf {v}}_i |$, and $\langle ... \rangle$ denotes the average over the system size. In the inset, we show the auto-correlation of the self-propulsion direction $\langle {\textbf {n}}_i(t) \cdot {\textbf {n}}_i(t = 0) \rangle$ for the same values of *J* and other parameters, as in Fig. 7a. With increase in *J*, time correlation of the self-propulsion direction ${\textbf {n}}_i$ increases. Hence, *J* increases the effective persistence time $\tau ^e_P$ of the disks. During head-on collision of two soft self-propelled disks, both the disks deform their shapes substantially and remain attached together on average for their persistence time scale $\tau _P = 1 / D_r$. On the other hand, if they collide with finite impact parameter, that is with an inter-particle distance less than the diameter of the disks, there is a finite probability for the disks to deform their shapes and move apart without much changing their self-propulsion direction^21^. Hence, the time required to remain attached decreases significantly for the soft disks, and their probability to collide with the other disks increases. Consequently, the number of collisions and the collision frequency increases with increase in *J* for the soft disks, as *J* increases the effective persistence time $\tau _P^e$. Hence, *J* favors MIPS for the soft disks. Interestingly, temporal correlation of the velocity ${\textbf {v}}_i$ of the disks increases with increase in *J*. In Fig. 7b, we show $\langle \hat{\text {v}}_i(t) \cdot \hat{\text {v}}_i(t = 0) \rangle$ for fixed $J = 2$ and for several values of disk softness parameter $\kappa$, shown in the legend. In the inset, $\langle {\textbf {n}}_i(t) \cdot {\textbf {n}}_i(t = 0) \rangle$ is shown for the same values of $\kappa$ and the other parameters, as in Fig. 7b. Time correlation of ${\textbf {n}}_i$ as well as ${\textbf {v}}_i$ is independent of the disk softness.

**Figure 8:**
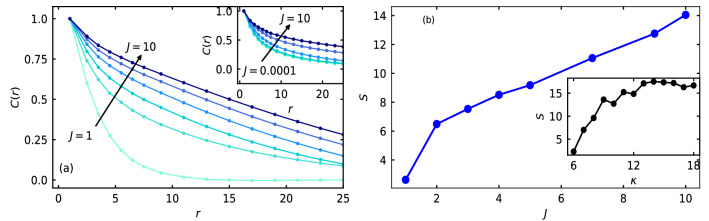
(**a**) *C*(*r*) of the soft disks at $\kappa = 7$ for several values of *J* in the range $J = [1, 10]$ as shown by the arrow, namely $J = 1$, $J = 3$, $J = 5$, $J = 7$, $J = 9$, and $J = 10$. The inset shows *C*(*r*) for $\kappa = 14$ and for *J* in the range $J = [0.0001, 10]$, as shown by the arrow. The other values of the parameters are $M = 0.05$ and $\text {P}_e = 125$. (**b**) Strength of spatial correlation of velocity *S*, quantified by integrating the *C*(*r*) data with respect to the *r* by fitting *C*(*r*) with the expression $m \frac{e^{- a r}}{r^b} + c$, with variation in *J* for fixed softness $\kappa = 7$. Inset shows *S* with variation in $\kappa$ for fixed $J = 2$.

We define the spatial correlation of velocity^37^ of the disks by the relation $C(r) = \frac{1}{N (N - 1)} \displaystyle {\sum _{i<j}} \hat{\text {v}}_i({\textbf {r}}_i) \cdot \hat{\text {v}}_j ({\textbf {r}}_j)$. Here, *r* is the magnitude of the inter-particle distance ${\textbf {r}}_{ij}$ between the disks at ${\textbf {r}}_{i}$ and at ${\textbf {r}}_{j}$. In Fig. 8a, we show *C*(*r*), time averaged over 50 steps in the interval of $\delta t = 1$, in the phase separated state for several values of *J* in the range [1, 10], as shown by the direction of the arrow, for the soft disks at $\kappa = 7$, $M = 0.05$, and $\text {P}_e = 125$.

In the inset, we show *C*(*r*) for increased hardness ($\kappa = 14$) over the full range of rotational inertia $J = [0.0001, 10]$, as the system phase separate at *J* as low as $J = 0.0001$. With increase in *J*, that is with increase in effective persistence time $\tau _P^e$, spatial correlation of velocity of the self-propelled disks increases both for the soft and substantially hard disks, which is consistent with the previous work in the respective limits^22,37,41,44^ Furthermore, the the strength of the correlation or the correlation length increases with disk hardness $\kappa$. However, spatial correlation of the self-propulsion direction ${\textbf {n}}_i$ has not been observed for our system. In Supplementary Fig. S6, we show the configuration of the system, colored according to the angle of velocity $\theta ({\textbf {v}}_i)$ (top row) and self-propulsion direction $\theta ({\textbf {n}}_i)$ (bottom row) with respect to the x-axis, for three different values of *J* in the phase separated state. To quantify spatial correlation of velocity or the spatial correlation length, we define a quantity *S*, which we call strength of the spatial correlation of velocity. To calculate *S*, we fit *C*(*r*) with the expression^41^: $m \frac{e^{- a r}}{r^b} + c$ and obtain the area under the fit curve using trapezoidal rule of numerical integration in the limit $r = 0.1$ to $r = 50$, that is $S = \int _0^{50} C(r + \Delta r) \Delta r$. Fix expression for *C*(*r*), which is inspired from a similar nature of spatial velocity correlation of hard disks in Ref. 41, is generalised in our work to incorporate the effect of *J* and $\kappa$. The generalized expression fits extremely well with our numerical data. In Fig. 8b, we show *S* as a function of *J* for fixed $\kappa = 7$, $M = 0.05$, and $\text {P}_e = 125$. In the inset, we show *S* as a function of disk softness $\kappa$, for fixed rotational inertia $J = 2$. The other parameter values are same as in Fig. 8b. Hence, strength of the spatial correlation of velocity or the spatial correlation length increases with increase in rotational inertia, while it decreases with increase in disk softness (decrease in $\kappa$). In Supplementary Fig. S7, we show the configuration, colored according to the angle of the velocity $\theta ({\textbf {v}}_i)$ with respect to the x-axis, for three different values of $\kappa$ at fixed *J*.

## Conclusion

We investigate the combined effect of rotational inertia and disk softness on the collective behavior of soft self-propelled inertial disks. Several important aspects have been addressed. We report the MIPS phase diagram in the space of rotational inertia of the disks and particle softness over a range of translational inertia and strength of self-propulsion, measured by the Peclet number. The phase boundary between homogeneous (no-MIPS) and MIPS state is found to shift towards larger disk softness with increase in rotational inertia. It was observed that in the limit of negligibly small rotational inertia, increase in softness disfavors MIPS^21^. We demonstrate that rotational inertial dynamics favors MIPS and opens up a new phase space for MIPS, as the system of much softer disks is found to exhibit MIPS in the limit of large rotational inertia. Shift in the phase boundary towards larger disk softness is shown to be the consequence of increased effective persistence of the self-propelled disks with increase in rotational inertia. Our analytical scaling Eq. (6) of the phase boundary are robust, as they are shown to agree with the numerical data over a large range of translational inertia of the self-propelled disks. Although we demonstrate the MIPS phase diagram in the space of rotational inertia and disk softness over a large range of translational inertia and self-propulsion strength, the nature of the non-equilibrium phase transition is an interesting open question of importance that can be attempted in future.

We demonstrate distinct structural and dynamical properties near and away from the phase boundary over a large range of translational inertia. The shape of the high density phase is found to be circular near the phase boundary, whereas it is found to be rectangular far away from the phase boundary, which is obtained either by increasing particle softness or rotational inertia. We quantify structural properties by measuring local area fraction and local orientational order of the disks, while dynamical properties are quantified by measuring local speed of the particles. Both structural and dynamical properties are found to remain constant in the central region of the high density phase, whereas they vary gradually near the peripheral region. Near the phase boundary, the peripheral region is shown to be much wider compared to narrow peripheral region away from the phase boundary. Also, the disks are found to infiltrate towards the center of the high density phase, as the local speed of the particles are finite in the high density phase, whereas away from the phase boundary, the particles are not found to move much inside the high density phase, as the local speed are nearly zero, as in conventional overdamped limit of hard self-propelled particles. The disk dynamics are quantified by measuring the fraction of particles retained in the central and the peripheral region of the high density phase. Spatial correlation of velocity of the disks in the high density phase is found to increase with both rotational inertia and particle hardness, whereas temporal correlation of the velocity of the disks in the high density phase is found to only vary with rotational inertia. Temporal correlation of the velocity of the disks in the high density phase is independent of disk softness. In this work, we consider a rectangular simulation box of fixed dimensions with periodic boundaries. It is likely that the shape and the boundaries of the simulation box would have a nontrivial impact on the properties of the clusters of the soft self-propelled disks, and this may be an interesting area for future studies.

We conjecture that our results should be observed in experiments with soft inertial balls with an appropriate self-propulsion mechanism^5^, in analogy with experiments of vibrated granular particles^25,45^. It would be crucial to perform such experiments in future work. We believe that our investigations are extremely important to extend the understanding of the collective properties of soft self-propelled particles and help in designing improved artificial self-propelled particles that could find its potential in several bio-medical applications^46,47^. Investigation of self-propelled particles with time dependent rotational inertia, as observed in several biological^36^ and synthetic^5^ active matter, would be interesting to perform.

## Supplementary Information

Supplementary Information 1.

Supplementary Information 2.

Supplementary Information 3.

Supplementary Information 4.

Supplementary Information 5.

## Data Availability

Data is available upon request to the corresponding author.
