# Supplementary material for: Collective behavior of soft self-propelled disks with rotational inertia
Source: Sci Rep. 2022 Dec 29;12:22563. doi: 10.1038/s41598-022-26994-2 (PMC9800414; doi:10.1038/s41598-022-26994-2)
Supplement: Supplementary file 1 — Supplementary Information 1. [file 41598_2022_26994_MOESM1_ESM.pdf]

# Supplemental Material: Collective behavior of soft self-propelled disks with rotational inertia

**Soumen De Karmakar**[1, 2, +], **Anshika Chugh**[1,2, ++], **and Rajaraman Ganesh**[1,2,*]

[1]Institute for Plasma Research, Bhat, Gandhinagar 382428, India
[2]Homi Bhabha National Institute, Training School Complex, Anushaktinagar, Mumbai 400094, India
[+]soumendekarmakar@gmail.com
[++]anshikachugh93@gmail.com
[*]ganesh@ipr.res.in

## Phase diagram

In Fig. S1, we show the phase diagram of the system of soft self-propelled disks in the $J-\kappa$ space in semi-log scale for three different values of translational inertia $M$, namely $M = 0.005$, $M = 0.05$, $M = 0.5$, spanning over two orders of magnitude. Peclet number is fixed at a small value $\mathrm{P}_e = 75$, compared to that in the main text at $\mathrm{P}_e = 125$. Green (triangles) and red (circles) markers denote the MIPS and the homogeneous phase respectively, as in the main text. The dashed and the solid lines are the analytic expression (Eq. 6 in the main text) for the phase boundary in the small and large $J$, respectively. Phase boundary shifts to larger $\kappa$ side with increase in $M$.

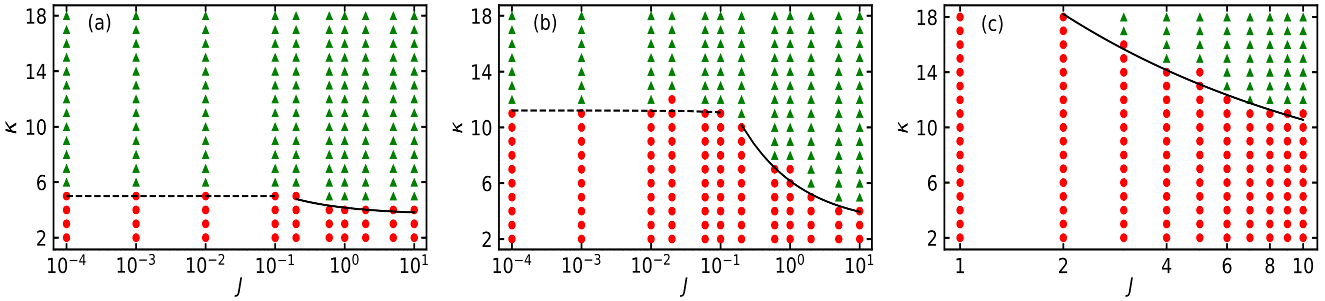


**Figure S1.** Phase diagram in the $J-\kappa$ space for (a) $M = 0.005$, (b) $M = 0.05$, and (c) $M = 0.5$ in semi-log scale for fixed $\mathrm{P}_e = 75$. Green (triangles) and red (circles) markers denote the MIPS and the homogeneous phase, respectively. Dashed and the solid lines are the analytic expression, Eq. 6 in the main text, in the small and large $J$ limit.

## Shape of the high density phase in the limit of large time

Configurations of the system at three different times, namely $t = 50$ (short time), $t = 100$ (intermediate time), and $t = 500$ (large time) for the fixed parameters $M = 0.05$, $J = 2$, $\kappa = 7$, $\mathrm{P}_e = 125$ is shown in Fig. S2. The particles in the high and low density phase are colored green and red, respectively. The shape of the high density phase remains circular even in the limit of large time $t = 500$.

## Structural and dynamical properties at small and large inertia

Structural and dynamical properties at small inertia $M = 0.005$ for $\kappa = 6$ (top row) and $\kappa = 18$ (bottom row) is shown in Fig. S3 at fixed $J = 1$ and $\mathrm{P}_e = 125$. In Figs. S3(a) and (d), local area fraction $\phi$ (left vertical axis) and the magnitude of local orientational order $q_6$ (right vertical axis) as a function of distance from the center of the high density phase is shown. The local speed v is shown in Figs. S3(c) and (f), and the configurations of the system, colored according to the magnitude of orientational order of the disks, are shown in Figs. S3(c) and (e). The system is near the phase boundary for $\kappa = 6$ and away from the phase boundary $\kappa = 18$ for the value of the other considered parameters. The magnitude of the local area fraction is much larger for $\kappa = 6$ compared to that for $\kappa = 18$, as the soft disks can significantly deform the other disks during collision.

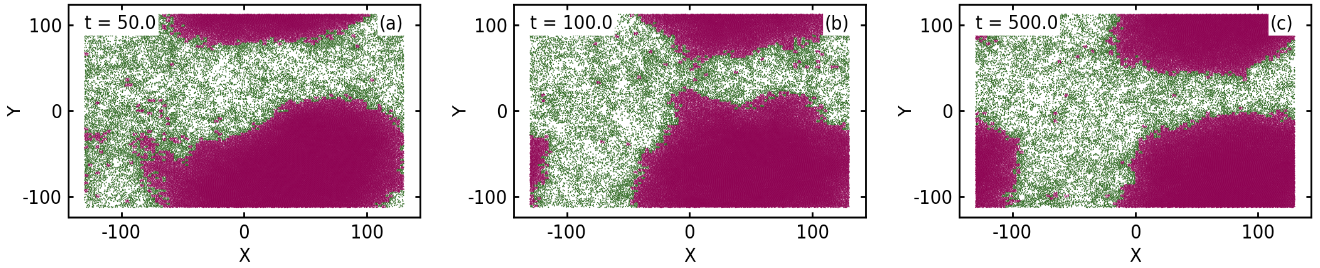


**Figure S2.** Configuration of the system at (a) $t = 50$ (short time), (b) $t = 100$ (intermediate time), (c) $t = 500$ (large time) for fixed $M = 0.05$, $J = 2$, $\kappa = 7$, and $P_e = 125$.

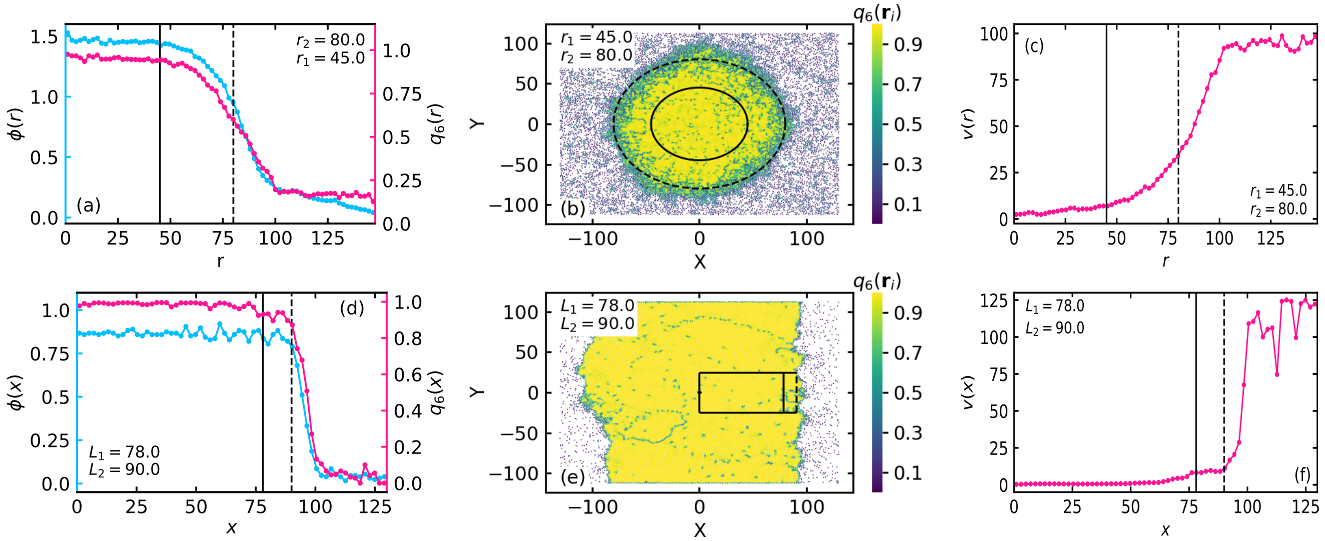


**Figure S3.** Local properties at small inertia $M = 0.005$ for $\kappa = 6$ (top row) and $\kappa = 18$ (bottom row) at fixed $P_e = 125$, $J = 1$. (a, d) Local area fraction $\phi$ (left vertical axis) and the magnitude of local orientational order $q_6$ (right vertical axis) as a function of distance from the center of the high density region. (b, e) Configurations, colored according to the magnitude of local orientational order $q_6$. (c, f) Local speed v as a function of distance from the center of the high density region.

Consequently, local orientational order for $\kappa = 6$ is slightly smaller than 1 compared to $q_6 = 1$ for $\kappa = 18$, as in $M = 0.05$ in the main text. The peripheral region $r_p \approx 35$ is wider for the soft disks at $\kappa = 6$, compare to the $r_p \approx 12$ for the hard disks at $\kappa = 12$. Magnitude of local speed in the central region for the hard disks ($\kappa = 18$) is $\mathrm{v} \approx 0$, whereas it is finite for the soft disks ($\kappa = 6$).

Local properties of the system at large inertia $M = 0.5$ for $J = 2$ (top row) and $J = 10$ (bottom row) are shown in Fig. S4 at fixed $\kappa = 18$, $P_e = 125$. For $M = 0.5$ the phase boundary is observed at large values of $\kappa$. The high density region is circular near the phase boundary ($J = 2$) even at large softness parameter $\kappa = 18$ for $M = 0.5$. With increase in $J$, the system moves away from the phase boundary, similar to that with increase in $\kappa$. The high density region is found to be rectangular in shape at large $J = 10$. In Fig. S4(a) and (d), local area fraction $\phi$ (left vertical axis) and magnitude of local orientational order $q_6$ is shown as a function of distance from the center of the high density phase. Configurations of the system, colored according to the magnitude of the local orientational order of the disks, are shown in Figs. S4(b) and (e). Local speed v is shown in Figs. S4(c) and (f). Value of the local area fraction near the phase boundary ($J = 2$) is similar in magnitude as that for $J = 10$, which is away from the phase boundary. The width of the peripheral region $r_p \approx 20$ for $M = 0.5$ is significantly smaller, compared to the width $r_p \approx 35$ for $M = 0.005$, near the phase boundary.

In Fig. S5, we compare the local speed v (left vertical axis) and the local area fraction $\phi$ (right vertical axis) for two different rotational inertia $J = 2$ (solid markers) and $J = 10$ (open markers) for fixed $M = 0.05$, $\kappa = 7$, and $P_e = 125$. Local speed and local area fraction both increases in the central region with increase in $J$, due to increase in effective persistence time $\tau_P^e$.

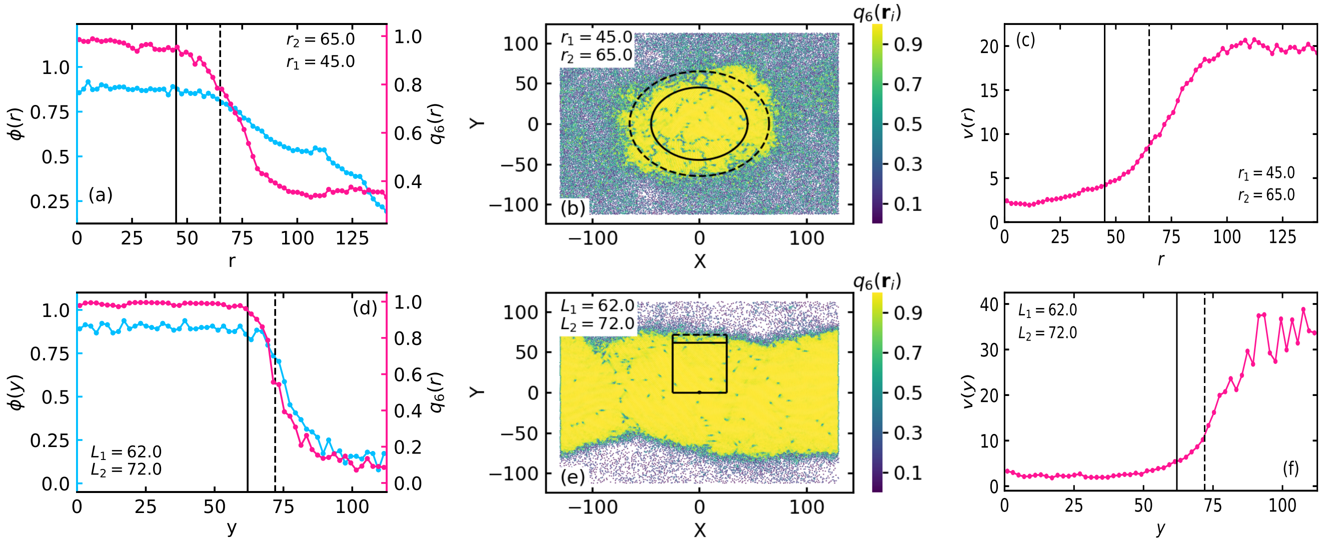


**Figure S4.** Local properties at large inertia $M = 0.5$ for $J = 2$ (top row) and $J = 10$ (bottom row) for fixed $\mathrm{P}_e = 125$, $\kappa = 18$. (a, d) Local area fraction $\phi$ (left vertical axis) and local orientational order $q_6$ (right vertical axis) from the center of the high density region. (b, e) Configuration of the system, colored according to the magnitude of orientational order of the disks $q_6(\mathbf{r}_i)$. (c, f) Local speed v of the disks from the center of the cluster.

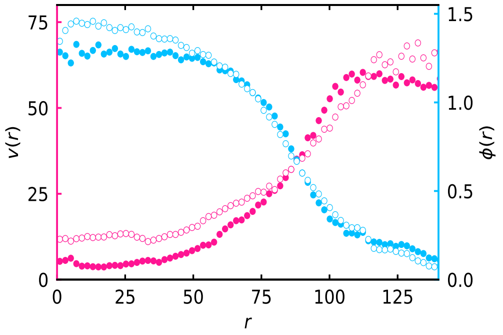


**Figure S5.** Local speed v (left vertical axis) and local area fraction $\phi$ (right vertical axis) from the center of the high density region of the circular cluster for $J = 2$ (filled circles) and $J = 10$ (open circles) for fixed $M = 0.05$, $\kappa = 7$, and $\mathrm{P}_e = 125$.

## Spatio-temporal correlation of velocity and self-propulsion direction

In Fig. S6, the configuration of the system for fixed $M = 0.05$, $\kappa = 7$, $\mathrm{P}_e = 125$, and for three different values of rotational inertia $J$, namely $J = 1$, $J = 5$ and $J = 10$, (from left to right) is shown. Colors in the top row correspond to the angle between the velocity $\mathbf{v}_i$ of the disks and the x-axis. Colors in the bottom row correspond to the angle between self-propulsion direction $\mathbf{n}_i$ of the disks and the x-axis. Velocity correlation length is found to increase with increase in $J$, however, no spatial correlation of the self-propulsion direction $\mathbf{n}_i$ is observed.

Configuration of the system for $\kappa = 7$ (left), $\kappa = 12$ (middle), and $\kappa = 18$ (right), for fixed $J = 2$, $M = 0.05$, $\mathrm{P}_e = 125$ is shown in Fig. S7. Colors denote the angle between $\mathbf{v}_i$ of the disks and the x-axis. With decrease in disk softness (increase in $\kappa$), spatial correlation of velocity of the disk increases.

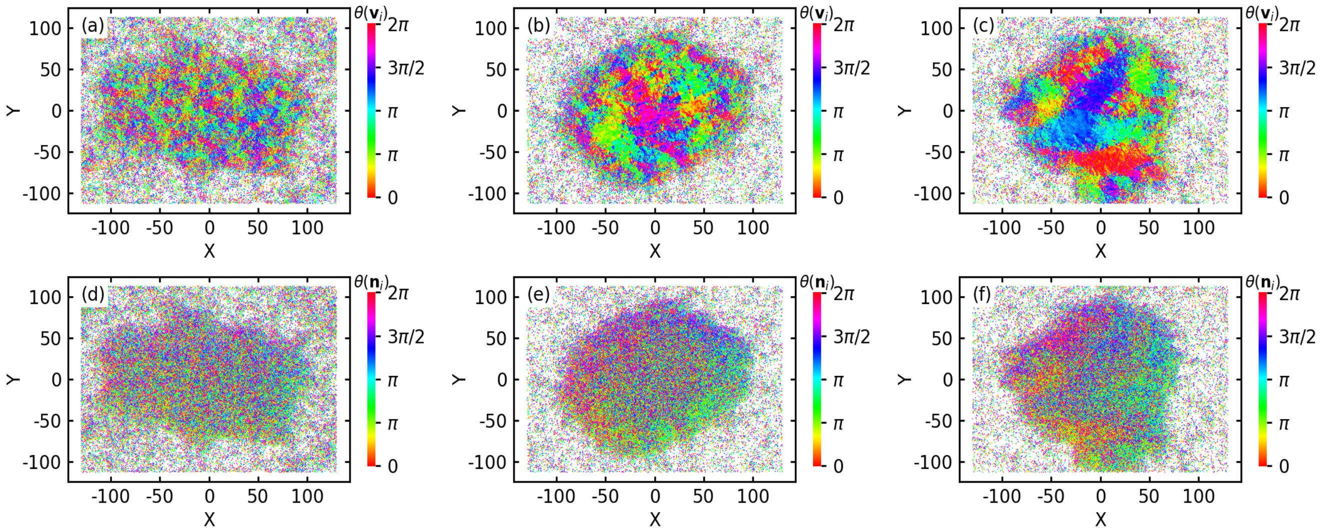


**Figure S6.** Configuration of the system of self-propelled disks for (a, d) $J = 1$, (b, e) $J = 5$, (c, f) $J = 10$ and for fixed $M = 0.05$, $\kappa = 7$, $P_e = 125$. Colors denote the angle of the velocity $\hat{v}_i$ (top row) and the self-propulsion direction $\mathbf{n}_i$ (bottom row) of the disks with respect to x-axis.

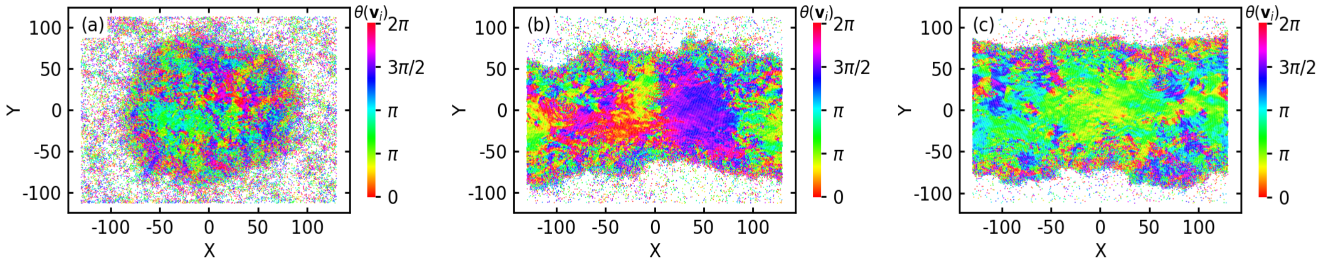


**Figure S7.** Configuration of the system of self-propelled disks for (a) $\kappa = 7$, (b) $\kappa = 12$, (c) $\kappa = 18$ and for fixed $J = 2$, $M = 0.05$, and $P_e = 125$. Colors denote the angle of the velocity $\hat{v}_i$ of the disks with respect to x-axis.

## Supplementary Movies

**Supplementary Movie 1** Dynamics of the self-propelled disks for $M = 0.05$, $J = 2$, $\kappa = 7$, and $\mathrm{P}_e = 125$. At an arbitrary initial time $t = t_1$, the center of the high density cluster is shifted at the center of the simulation box of size $L_x \times L_y \approx 520 \times 450\sigma^2$. At $t = t_1$, the disks in the central region are colored green and the peripheral region are colored yellow, and the other disks are colored purple.

**Supplementary Movie 2** Dynamics of the self-propelled disks for $\kappa = 18$ (hard particle limit). The other value of the parameters are same as that in Supplementary movie 1. At an arbitrary initial time $t = t_1$, the center of the high density cluster is shifted to the center of the simulation box and disks are colored as in Supplementary movie 1.

**Supplementary Movie 3** Dynamics of the self-propelled disks for large $J = 10$. The value of the other parameters are same as that in Supplementary movie 1. At an arbitrary initial time $t = t_1$, the center of the high density cluster is shifted at the center of the simulation box and the disks are colored as in Supplementary movie 1.

**Supplementary Movie 4** Time evolution of the spatial velocity correlation of the self-propelled disks for $M = 0.05$, $J = 10$, $\kappa = 7$, and $\mathrm{P}_e = 125$. Colors denote the angle of the velocity $\hat{v}_i$ of the disks with respect to x-axis.
